# Ultrasound evaluation of diaphragmatic function in patients with idiopathic pulmonary fibrosis: a retrospective observational study

**DOI:** 10.1186/s12931-023-02577-1

**Published:** 2023-10-28

**Authors:** Jules Milesi, Alain Boussuges, Paul Habert, Julien Bermudez, Martine Reynaud-Gaubert, Stéphane Delliaux, Fabienne Bregeon, Benjamin Coiffard

**Affiliations:** 1 Department of Respiratory Medicine and Lung Transplantation, Assistance Publique-Hôpitaux de Marseille (AP-HM), Hôpital Nord, Aix-Marseille University, Chemin des Bourrely, 13015 Marseille, France; 2 Pulmonary Function Testing Laboratory, APHM, Aix-Marseille University, Marseille, France; 3 Department of Radiology, APHM, Aix-Marseille University, Marseille, France; 4 LIIE, Aix-Marseille University, Marseille, France; 5 CERIMED, Aix-Marseille University, Marseille, France

**Keywords:** Diaphragm, Ultrasonography, Idiopathic pulmonary fibrosis, Lung diseases, Physiology, Respiratory physiological phenomena, Musculoskeletal physiological phenomena, Tomography, X-Ray computed, Respiratory function tests

## Abstract

**Introduction:**

The diaphragm function assessed by ultrasound has been well-studied in COPD, asthma, and intensive care. However, there are only a few studies on diffuse interstitial lung disease, while dyspnea and quality of life are major issues in the management that may depend on the diaphragm.

**Methods:**

We retrospectively included idiopathic pulmonary fibrosis (IPF) patients followed in our center (Marseille, France) between January 2020 and February 2023 who underwent diaphragmatic ultrasound. Our objectives were to describe the diaphragmatic function of IPFs compared to healthy controls and to correlate with clinical, functional, and lung density on CT-scan.

**Results:**

24 IPF patients and 157 controls were included. The diaphragmatic amplitude in IPF was increased at rest (median of 2.20 cm vs 1.88 cm on the right, p < 0.007, and 2.30 cm vs 1.91 cm on the left, p < 0.03, in IPF and controls respectively) and decreased in deep breathing (median of 4.85 cm vs 5.45 cm on the right, p < 0.009, and 5.10 cm vs 5.65 cm on the left, p < 0.046, in IPF and controls respectively). Diaphragmatic thickness was significantly reduced at rest on the right side (median of 1.75 mm vs 2.00 mm, p < 0.02, in IPF and controls respectively) and in deep breathing on both sides compared to controls (mean of 3.82 mm vs 4.15 mm on the right, p < 0.02, and 3.53 mm vs 3.94 mm, on the left, p < 0.009, in IPF and controls respectively). Diaphragmatic amplitude in deep breathing was moderate to strongly correlated with FVC, DLCO, and 6MWT and negatively correlated with the dyspnea and lung density on CT scan.

**Conclusion:**

The diaphragmatic amplitude and thickness were impaired in IPF compared to controls. Diaphragmatic amplitude is the parameter best correlated with clinical, functional, and lung density criteria. Further studies are needed to determine if diaphragmatic amplitude can be a prognostic factor in IPF.

## Introduction

In interstitial lung disease (ILD), dyspnea and quality of life are major issues in patient care [1–3]. Several mechanisms can explain dyspnea, but among them, the diaphragmatic function is an important determinant because it is responsible for about 2/3 of the tidal volume at rest [4, 5]. The causes of diaphragmatic dysfunction in ILD may be multiple, namely, mechanical disadvantage due to restriction, chronic hypoxia, chronic inflammation, corticosteroids use, or exercise deconditioning [6–8].

Different methods are used to assess diaphragmatic function including imaging by fluoroscopy, computed tomography to assess both the structure and function of the diaphragmatic muscle or the measurement of trans-diaphragmatic pressures, stimulation of the phrenic nerve, and electromyography exclusively evaluating neuromuscular function. However, all these methods have interpretation limits, and sometimes technical constraints related to their invasive nature [9–12]. Thus, diaphragmatic ultrasound has a primordial place in this indication because of its accessibility, its non-invasive nature, and its inter- and intra-observer reproducibility [13–19].

The measurement of diaphragmatic function by ultrasound has already been studied in many lung diseases such as asthma [19], COPD [20–22], cystic fibrosis [23], or even in intensive care patients with respiratory failure [24–26]. Norms of ultrasound diaphragmatic function in healthy subjects have recently been recently published by Boussuges et al. [29, 30]. However, there are only a few studies on ultrasound assessment of diaphragmatic function in ILD [27, 28, 31, 32], and they each present some limits: small population of patients, a very heterogeneous panel of ILD, no correlation with fibrosis extension on CT scan, or no correlation to the muscular mass of the patients. Thus, further studies are needed to assess diaphragm function in ILD, particularly in IPF.

This study aimed to describe the diaphragmatic structure and function by ultrasound in a homogeneous population of IPF and to compare them with healthy subjects.

## Methods

### Study design and population

We conducted a retrospective monocentric study at the North University Hospital of Marseille, France. All patients with IPF consecutively evaluated between December 2020 and February 2023 for lung transplantation in the Department of respiratory medicine were included. Patients benefited from a systematic evaluation of the diaphragmatic function by ultrasound in the pulmonary function test laboratory in the pre-transplant assessment.

Participants had a diagnosis of IPF based on clinical, biological, functional, CT scan, and possibly histological criteria accordingly to the 2018 ATS/ERJ criteria [33]. Patients with stable disease with no therapeutic change in the last 3 months were included. Were excluded patients with a confounding pulmonary pathology such as COPD, cystic fibrosis or other bronchi’s dilatation, autoimmune diseases with arguments for clinical or biological muscle damage, myopathy, an active infection, a history of upper abdominal surgery or thoracic surgery (other than for the diagnosis of IPF), exacerbation or rehabilitation of less than 2 months.

Healthy subjects from a previously published study by Boussugues et al. [30] that described normal values of diaphragm thickness were used as control cases. Only subjects who performed diaphragm ultrasounds and PFTs in our center (North Hospital University of Marseille) were included (82 men and 75 women).

The Institutional Review Board of the French learned society for respiratory medicine-Société de Pneumologie de Langue Française—approved the protocol (CEPRO 2022-033bis), and a notice of information and non-objection was given to all participants according to French law.

### Clinical data collected

For all the subjects, we collected the following data carried out in clinical routine, the closest to the diaphragmatic evaluation: age, sex, BMI, smoking status, dyspnea according to the mMRC (modified Medical Research Council) scale, 6-min walk test (6MWT), oxygen supplementation, co-morbidities, treatments used such as corticosteroids or immunosuppressants. All participants benefited from spirometry, plethysmography, and diffusion analysis when available (Ilmeter 1304; Masterlab Jaeger, Wurzberg, Germany).

### Diaphragm ultrasound measurements

The diaphragm ultrasound measurements were performed with the patient in a seated position, on the right and the left side, from the same ultrasound device (Vivid S60N, GE Healthcare, Milwaukee, Wl, USA). A single experienced operator (AB) performed all the measurements (IPF and controls). To strengthen the accuracy of the results, all measurements were averaged from at least three different breathing cycles.

Measurement of the diaphragmatic excursion (or amplitude) was carried out using a cardiac probe placed between the midclavicular and anterior axillary lines, in the subcostal area, and directed medially, cranially, and dorsally, so that the ultrasound beam reached perpendicularly the posterior third of the right hemidiaphragm. On the left side, a subcostal or low intercostal probe position was chosen between the anterior and mid-axillary lines to obtain the best imaging of the hemidiaphragmatic dome. Diaphragm movements were recorded in M-mode. Ultrasonographic measurements were performed during quiet breathing (QB), deep breathing (DB), and voluntary sniffing (VS). This maneuver began at the end of normal expiration, and the subjects were asked to breathe in as deeply as they possibly could.

Diaphragmatic thickness was measured using a high-frequency linear 9L probe placed on the diaphragm insertion on the rib cage between the anterior axillary and mid-axillary lines, according to a previously published method [30]. Measurements were taken in B-Mode, and the diaphragm was identified at the level of the thoracoabdominal junction as a hypoechoic structure with a hyperechoic line in its center and surrounded by 2 hyperechoic structures, the pleural and the peritoneal layers. The diaphragmatic thickness was measured as the distance from the middle of the pleural membrane to the middle of the peritoneal membrane in expiration (functional residual capacity) and in inspiration during QB and DB. The thickening fraction was measured according to the following formula: (thickness at end-expiratory (Tee)—thickness at end-inspiratory during QB or DB (Tei or Tei max))/thickness at end-expiratory (Tee) × 100.

### Computed tomography (CT) measurements

We analyzed thoracic CT scans closest to the diaphragmatic ultrasound evaluation in IPF patients exclusively. All thoracic CTs were performed according to the following parameters: 120 kV and 1 mAs/kg with care dose modulation and reconstruction in joint slices of 1:1 mm. Doses were adjusted manually according to the patient template: 100 kV if they weighed < 60 kg, if above 120 kV. The thoracic CT scans were acquired during breath-hold inspiration from the adrenal glands to the neck and at the end of forced expiratory flow. The total lung volume was recorded from the CT inspiratory volume using the post-treatment station, Thoracic VCAR (GE Healthcare). CT scans were performed on various systems (Revolution EVO, Revolution Maxima, Revolution Frontier, Revolution HD, Revolution CT, GE Healthcare, WI, USA). Analyses of the lung parenchyma density were performed using dedicated 3D analysis software (3D Slicer, https://www.slicer.org). From the parenchymal window, the lungs were segmented and reconstructed in 3D by selecting the Hounsfield unit (HU) from − 1024 to − 350. The trachea and main bronchi were excluded. The densitometric analysis consisted in quantifying the voxels on the whole lungs by HU. The threshold of -600 HU was used to calculate fibrosis (High Attenuation Area, HAA: % of voxel > − 600 HU). The voxel quantification histogram by HU was extracted and the curve flattening coefficient (Kurtosis) and the asymmetry coefficient (Skewness) were calculated (because linked to the quantity of voxel with HU between − 600 and − 350 and therefore to fibrosis) [34].

### Statistics analysis

A descriptive analysis was performed on the IPF group and the healthy controls. Continuous variables are expressed in median and interquartile or mean and standard deviation, depending on the distribution (Shapiro–Wilk test), and qualitative variables are expressed in numbers and percentages. Analyzes were performed to compare patient characteristics and ultrasound measurements between groups. Qualitative parameters were compared using Chi-square tests. Quantitative parameters were compared using a student’s test or a Mann–Whitney-Wilcoxon non-parametric test depending on the distribution. Correlation tests between the diaphragmatic measurements, the clinical and functional pulmonary data, and the CT scan measurements were performed by the Pearson method.

All tests are two-sided. A p < 0.05 was considered significant. The analysis was performed using version 4.2.1 (2022-06-23) of the R software (R Core Team (2022). R: A language and environment for statistical computing. R Foundation for Statistical Computing, Vienna, Austria. URL https://www.R-project.org/).

## Results

### Baseline characteristics

During the study period, 24 IPF patients met the inclusion criteria and were compared to 157 control patients. The patient and control characteristics are in Table 1**.**

**Table 1:** Baseline characteristics

	Patients	Controls	p
No of patients, n	24	157	
Age (year)	66 ± 6	50 ± 17	< 0.001
Sex (female), n (%)	5 (21)	75 (48)	0.02
Height (cm)	171 ± 8	170 ± 9	0.26
Weight (kg)	77 ± 16	72 ± 13	0.16
Body mass index (kg/m^2^)	25.6 ± 3.7	24.8 ± 3.7	0.22
Smoking history, n (%)	21 (87)		
Pack-years	24 ± 16		
Comorbidities, n (%)			
Diabetes	5 (21)		
High blood pressure	4 (17)		
Cardiovascular disease	11 (46)		
UIP pattern on CT-scan, n (%)			
Definite	12 (50)		
Probable	11 (46)		
Unclassifiable	1 (4)		
Pulmonary hypertension, n (%)	8 (33)		
sPAP (mmHg)	38 [33; 45]		
mPAP (mmHg)	27 ± 6		
PVR (Wood)	2.9 [2.1; 4.2]		
Dyspnea (mMRC score)	2 [1; 2]		
O2 supplementation, n (%)	8 (33)		
Specific fibrosis therapy, n (%)	21 (87)		
Nintedanib, n (%)	17 (77)		
Pirfenidone, n (%)	19 (83)		
Systemic corticosteroids, n (%)	4 (17)		
Dosing (mg)	7.5 [5; 22]		
Lung function test			
FEV1 (L)	2.3 ± 0.6	3.2 ± 0.9	< 0.001
FEV1 (% pred)	78 ± 20	100 ± 14	< 0.001
FVC (L)	2.7 ± 0.8	4.0 ± 1.1	< 0.001
FVC (% pred)	72 ± 21	103 ± 12	< 0.001
FEV1/FVC ratio (%)	84 ± 6	81 ± 6	0.02
TLC (L)	4.4 ± 1.0		
TLC (% pred)	69 ± 15		
DLCO (% pred)	41 ± 12		
Six minutes walking test (m)	524 [455; 553]		
Blood biology			
Albumin (g/L)	41 [38; 44]		
CPK (IU/L)	79 ± 33		
Creatinine (µmol/L)	71 ± 18		
CRP (mg/L)	3 [2; 9]		
ANA, n (%)	15 (62)		
ANCA, n (%)	2 (9)		
Time between diagnosis and PFT (days)	1604 ± 1131		
Time between diagnosis and US (days)	1632 ± 1137		
Time between PFT and US (days)	13 [0; 64]		
Time between diagnosis and CT (days)	1555 ± 1147		
Time between PFT and CT (days)	0 [-97; 3]		

*UIP* usual interstitial pneumonia, *PAP* pulmonary arterial pressure, *PVR* pulmonary vascular resistance, *mMRC* modified Medical Research Council, *ANA* antinuclear antibody, *PFT* pulmonary function test, *US* ultrasound

### Diaphragm measurements

Measurements of the diaphragmatic structure and function by ultrasound of IPF patients and controls are in Table 2. The diaphragmatic amplitude at rest of IPF patients was significantly greater than controls on the right, p < 0.007, and on the left side, p < 0.03. The maximum amplitude was, however, significantly lower in IPF patients bilaterally: on the right, p < 0.009, and on the left side, p < 0.046. The diaphragm thickness at rest (Tee) was lower in IPF patients compared to controls, significantly on the right side, p = 0.02, and p = 0.06 on the left side. The diaphragm thickness at maximum inspiration (Tei max), was lower in IPF patients bilaterally: on the right, p = 0.02, and on the left side, p = 0.009. Amplitude during voluntary sniffing (Amp sniff), thickness at end-inspiration (Tei), and thickening fraction (TF and TF max) were not significantly different between IPF and controls.

**Table 2:** Comparison of diaphragmatic function of IPF patient’s vs controls

	Patients, n: 24	Controls, n: 157	p
Right side			
Amplitude (cm)	2.20 [1.80; 2.50]	1.88 [1.68; 2.15]	0.007
Amplitude max (cm)	4.85 [4.17; 5.82]	5.45 [4.80; 6.07]	0.009
Amplitude sniff (cm)	2.60 [2.30; 2.70]	2.40 [2.07; 2.87]	0.43
Tee (mm)	1.75 [1.60; 1.92]	2.00 [1.70; 2.30]	0.02
Tei (mm)	2.40 [2.20; 2.90]	2.60 [2.30; 3.10]	0.29
Tei max (mm)	3.82 ± 0.59	4.15 ± 0.81	0.02
Ratio Tei/Tei max	0.66 ± 0.13	0.65 ± 0.11	0.51
TF (%)	38 [26; 53]	31 [21; 43]	0.10
TF max (%)	121 [85; 138]	100 [82; 135]	0.29
Left side			
Amplitude (cm)	2.30 [1.95; 2.40]	1.91 [1.60; 2.39]	0.03
Amplitude max (cm)	5.10 [3.95; 5.85]	5.65 ± 0.96	0.046
Amplitude sniff (cm)	2.65 [2.12; 3.15]	2.50 [2.13; 2.95]	0.49
Tee (mm)	1.70 [1.55; 1.90]	1.80 [1.60; 2.10]	0.06
Tei (mm)	2.20 [2.10; 2.45]	2.40 [2.00; 2.83]	0.13
Tei max (mm)	3.53 ± 0.63	3.94 [3.35; 4.52]	0.009
Ratio Tei/Tei max	0.64 ± 0.13	0.62 ± 0.09	0.53
TF (%)	31 [25; 38]	28 [20; 38]	0.16
TF max (%)	111 [91; 141]	108 [88; 137]	0.94

*Tee* end-expiratory thickness, *Tei* end-inspiratory thickness, *TF* thickening fraction

### Correlations between diaphragm measurements and pulmonary function

**Fig. 1:**
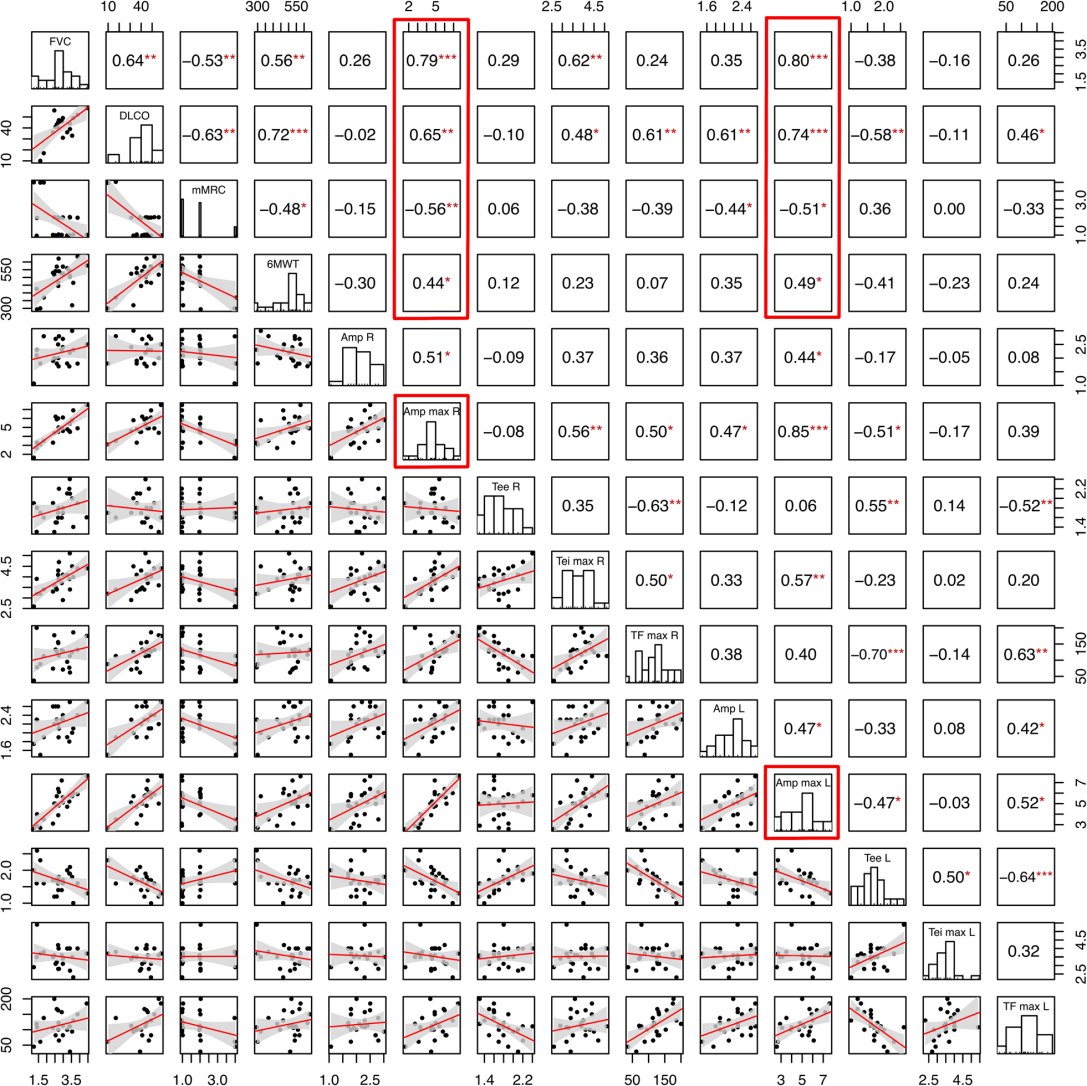
Pearson correlation matrix between FVC, DLCO, dyspnea evaluated with the mMRC scale, 6MWT and diaphragmatic ultrasound parameters in IPF patients. *Amp* amplitude, *Tee* end-expiratory thickness, *Tei* end-inspiratory thickness, *TF* thickening fraction

The results are depicted in Fig. 1 as a correlation matrix. Right and left maximal diaphragmatic amplitude were positively correlated with FVC (r = 0.79, p < 0.001 and r = 0.80, p < 0.001, respectively), DLCO (r = 0.65, p < 0.01 and r = 0.74, p < 0.001, respectively), and 6MWT (r = 0.44, p < 0.05 and r = 0.49, p < 0.05, respectively), and negatively correlated with mMRC score (r = − 0.56, p < 0.01 and r = − 0.51, p < 0.05, respectively). Right and left diaphragmatic thickening fraction were positively correlated with DLCO (r = 0.61, p < 0.01 and r = 0.46, p < 0.05, respectively) but not with FVC.

### Correlations between diaphragm function and lung density

**Fig. 2:**
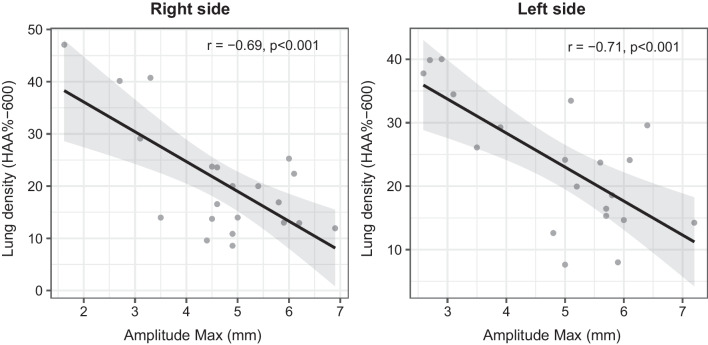
Pearson correlation between HAA%-600 and maximal diaphragmatic amplitude in deep breathing in IPF patients

The analyses of the lung parenchyma density in IPF patients are presented in Table 3. Pulmonary fibrosis evaluated by the density of the pulmonary parenchyma with the % of voxels greater than − 600 HU (HAA%− 600) was negatively correlated with the maximal diaphragmatic amplitude in DB on the right (r = − 0.69, p < 0.001) and on the left side (r = − 0.71, p < 0.001), Fig. 2.

**Table 3:** Lung density measurements on thoracic CT-scan

Patients (n: 24)	Whole lung	Right lung	Left lung
HAA-600 (%)	22 ± 10	21 ± 11	23 ± 10
Mean (HU)	− 722 ± 77	− 730 ± 83	− 714 ± 78
Kurtosis	5.1 ± 3.2	5.9 ± 3.8	4.7 ± 3.4
Skewness	1.96 ± 0.55	2.08 ± 0.63	1.88 ± 0.56

*LAA* low attenuation area, *HAA* high attenuation area

## Discussion

This study demonstrates that diaphragmatic function assessed by ultrasound in IPF patients showed significant differences compared to healthy controls. First, we found that diaphragmatic amplitude at rest in IPF was significantly increased compared to controls. This finding might be due to diaphragmatic compensation, indicating the need for greater muscular work to maintain the same degree of hematosis. Such muscle work at rest can lead to a decrease in reserve strength and faster dyspnea. Second, a decrease in the maximal diaphragmatic excursion, which is consistent with available evidence-based medicine [27, 28, 31, 32], probably because, during exercise, the diaphragmatic capacities are limited by the lung, so the amplitude is weaker than in healthy subjects. These results are most likely the consequence of an alteration of the thoraco-pulmonary compliance and an impaired elastic recoil related to pulmonary fibrosis that are responsible for a mechanical constraint on the diaphragm. And last, like Santana et al. [27, 28], we found a lower diaphragm thickness in DB in IPF patients. However, the diaphragm thickness was also lower in QB, which is inconsistent with previous studies. These differences could be explained by the fact that other studies have focused on a heterogeneous panel of ILDs [27, 28], very few patients [31], or patients with less impaired lung function [32]. In our study, all patients were evaluated for lung transplantation in the setting of severe disease that could explain muscle deconditioning and diaphragmatic atrophy. Indeed, diaphragmatic atrophy might be induced by a chronic muscle injury related to work overload or even hypoxia, malnutrition, age, systemic inflammation, exercise deconditioning, or corticosteroid use [6–8, 35]. Interestingly, even though diaphragm thickness was lower in DB and QB in our cohort, there was no difference in thickening fraction between patients and controls, meaning there was no muscular (or intrinsic) diaphragm dysfunction. On the contrary, Santana et al. [27, 28] showed a decreased thickening fraction in patients probably due to more advanced disease.

The diaphragm amplitude in DB was in our study well correlated with clinical features: positive correlation with FVC, DLCO, 6MWT, and negative correlation with dyspnea. Previous studies found also that amplitude was strongly and positively correlated with FVC and DLCO, which are strong predictive factors of mortality in IPF and ILDs [27, 28, 31]. Correlation with dyspnea at exercise (6MWT) and at rest (mMRC scale) could be explained by the increase of diaphragmatic work because of lung stiffness, to maintain the same level of exercise, eliciting early onset breathlessness [3]. The relationship between pulmonary volume and diaphragm excursion is debated and controversial in the literature. Some studies found a linear relationship between inspiratory lung volume and diaphragmatic excursion [36, 37], whereas others found only a weak correlation between lung volume and diaphragm amplitude [16, 38]. We think it is because inspiratory lung volumes are not only determined by diaphragmatic mobility but also by the recruitment of extra diaphragmatic muscles and thoraco-pulmonary compliance [16, 27, 39]. Walterspacher et al. showed a global respiratory muscle strength remains preserved in ILD patients [40] but not diaphragm force, which could explain the correlation between lung volumes and diffusion capacity with diaphragmatic function in our cohort. Indeed, the DLCO is positively correlated with the diaphragm function probably because it reflects the extension of the fibrosis as FVC and lung stiffness [7], but also because impaired diaphragm function may hinder ventilation throughout exercise causing additional mismatch on the ventilation to perfusion ratio.

Finally, like the other works studying diaphragm in IPF or ILD [27, 28, 31], we showed that diaphragmatic amplitude in DB was the ultrasound parameter most correlated with clinical and functional data. Nevertheless, we may notice differences between studies depending on the patient’s disease severity. The thickening fraction was altered compared to controls and correlated to clinical and functional outcomes only for Santana et al. [27, 28] where patients had the worse lung function. A decrease in diaphragm amplitude and a correlation to clinical and functional outcomes were shown in all studies [27, 28, 31], except for Kismet et al. [32], who had less impaired lung function and found no correlation. Thus, diaphragmatic amplitude seems to be reduced and correlated earlier with clinical and functional outcomes of IPF patients than the thickening fraction. In our opinion, the amplitude better reflects extrinsic diaphragmatic dysfunction related to lung fibrosis whereas the thickening fraction better reflects intrinsic (or muscle) diaphragmatic dysfunction secondary to chronic muscle injury. We found that DLCO was positively correlated with the thickening fraction but not FVC. The pathophysiology is multifactorial and may be related to pulmonary hypertension that was systematically present in our cohort of IPF patients but may also involve ventilation/perfusion phenomenon. Since the insertion of the diaphragm pillar is in West’s zone 3, where lung perfusion is greater than ventilation [41], even a small muscle weakness of the diaphragm could lead to decreased recruitment of pulmonary vessels and impaired diffusive lung capacity. Another way of putting it is that a decrease in the thickening fraction increases the ventilation/perfusion mismatch and can change the DLCO.

Regarding lung density assessed by CT, we found a negative correlation between the proportion of voxels greater than -600HU (as a surrogate of lung fibrosis) and the diaphragmatic amplitude in DB. This highlights the relationship between thoraco-pulmonary compliance and a mechanical constraint on the diaphragm in IPF patients. Kismet et al. [32] also analyzed the pulmonary parenchyma with the Total Fibrosis Score (TFS) but found no link with diaphragmatic function, perhaps due to a less precise assessment of fibrosis or patients with less severe lung disease.

This study has several strengths. Our cohort of IPF patients was compared to a large cohort of healthy controls used to define the normal value of diaphragm measurements by ultrasound [30]. For technical reasons, the diaphragm ultrasound of Santana et al. [27, 28], Kismet et al. [32], and Boccatonda et al. [31], was only analyzed on the right side. To our knowledge, this is the first bilateral ultrasound evaluation in ILDs. In addition, correlations were made with clinical and functional parameters, but also measurement of lung density was assessed by CT scan to obtain an objective assessment of the extent of fibrosis lesions.

Several limitations should nevertheless be noted. This is a monocentric study including a small number of IPF patients. Due to the retrospective nature of the study, data to quantify the patient's peripheral muscle mass were not available to assess the extent of the global muscle weakness. However, the cohort of IPF patients in this study had normal blood albumin levels, BMI around 25, and was not strongly deconditioned (median 6MWT at 524 m). We, thus, may hypothesize that patients had no strong sarcopenia. And last, we had no access to quality-of-life data, an essential element to characterize the impact of the disease in IPF patients [1–3].

## Conclusion

Our study shows that diaphragmatic amplitudes in QB and DB are altered in IPF compared to controls probably because of the change of the thoraco-pulmonary compliance responsible for a mechanical constraint on the diaphragm. Moreover, predictors of mortality such as FVC and DLCO, clinical outcomes such as 6MWT and dyspnea, and lung density are well correlated with the diaphragmatic amplitude in DB. Further studies are needed to know if the diaphragmatic amplitude could be a prognostic factor in IPF and is associated with exacerbations, hospitalizations, or mortality.

## Data Availability

Anonymized data will be shared upon request from any qualified investigator for purposes of replicating procedures and results.

## Abbreviations

ILD: Interstitial lung disease
mMRC: Modified-Medical Research Council
6MWT: 6 Minutes walk-test
IPF: Idiopathic pulmonary fibrosis
QB: Quiet breathing
DB: Deep breathing
VS: Voluntary sniffing
Amp: Amplitude
Tee: End-expiratory thickness
Tei: End-inspiratory thickness
Teimax: End-inspiratory maximal thickness
Amp Sniff: Amplitude during voluntary sniffing
TFS: Total Fibrosis Score
TF: Thickening fraction
LAA: Low attenuation area
HAA: High attenuation area
UIP: Usual interstitial pneumonia
PAP: Pulmonary arterial pressure
PVR: Pulmonary vascular resistance
ANA: Antinuclear antibody
PFT: Pulmonary function test
US: Ultrasound
